# DNA-Based Fluorescent Nanoprobe for Cancer Cell Membrane Imaging

**DOI:** 10.3390/molecules29010267

**Published:** 2024-01-04

**Authors:** Xiaoqiao Wu, Xinjia Shuai, Kunhan Nie, Jing Li, Lin Liu, Lijuan Wang, Chengzhi Huang, Chunmei Li

**Affiliations:** 1 Department of Basic Medicine, Shangqiu Medical College, Shangqiu 476100, China; wxqiao1123@163.com; 2 Key Laboratory of Luminescence Analysis and Molecular Sensing (Southwest University), Ministry of Education, College of Pharmaceutical Sciences, Southwest University, Chongqing 400715, China; s19971102@email.swu.edu.cn (X.S.); n17784292276@email.swu.edu.cn (K.N.); lj981028@email.swu.edu.cn (J.L.); liul0077@163.com (L.L.); chengzhi@swu.edu.cn (C.H.)

**Keywords:** DNA nanomaterials, cell membrane, fluorescence imaging

## Abstract

As an important barrier between the cytoplasm and the microenvironment of the cell, the cell membrane is essential for the maintenance of normal cellular physiological activities. An abnormal cell membrane is a crucial symbol of body dysfunction and the occurrence of variant diseases; therefore, the visualization and monitoring of biomolecules associated with cell membranes and disease markers are of utmost importance in revealing the biological functions of cell membranes. Due to their biocompatibility, programmability, and modifiability, DNA nanomaterials have become increasingly popular in cell fluorescence imaging in recent years. In addition, DNA nanomaterials can be combined with the cell membrane in a specific manner to enable the real-time imaging of signal molecules on the cell membrane, allowing for the real-time monitoring of disease occurrence and progression. This article examines the recent application of DNA nanomaterials for fluorescence imaging on cell membranes. First, we present the conditions for imaging DNA nanomaterials in the cell membrane microenvironment, such as the ATP, pH, etc. Second, we summarize the imaging applications of cell membrane receptors and other molecules. Finally, some difficulties and challenges associated with DNA nanomaterials in the imaging of cell membranes are presented.

## 1. Introduction

The cell membrane is a semi-permeable membrane that acts as a natural barrier to prevent extracellular substances from freely entering cells, exhibiting a high selectivity, permeability, and fluidity [1,2,3]. Lipids and proteins are the primary components of the cell membrane. Lipid bilayers can self-assemble from membrane-forming lipids containing a hydrophilic head and two hydrophobic alkyl tails [4]. Consequently, the cell membrane functions as a barrier and gatekeeper to control the transport of information and substances within the cell. In addition, it plays a crucial role in protecting cells from extracellular influences, maintaining intracellular homeostasis, and regulating cell functions and behaviors [5,6]. In addition, it is of great importance for the investigation of the molecular mechanisms underlying diverse biological processes in order to achieve the imaging or functional regulation of the cell membrane microenvironment and receptors. 

Currently, X-ray tomography [7], magnetic resonance imaging [8], and ultrasound [9] have been widely applied to the study of disease development, but they cannot achieve the real-time monitoring of multiple molecules and physiological parameters in cells, preventing the timely acquisition of pertinent information such as disease progression [10]. However, some other imaging techniques can overcome the aforementioned drawbacks, such as biological imaging technology, which provides a more direct method of revealing multidimensional information from biomolecules and cells to organs and living individuals [11]. Cellular fluorescence imaging is significant for biosensing and early disease diagnosis owing to the following characteristics. Firstly, it has the advantages of visual biodistribution, real-time information feedback, and ease of operation [12]. Secondly, fluorescence imaging enables the simultaneous detection of multiple targets and the real-time imaging of cell-specific molecular targets, pathways, and physiology, enabling early accurate diagnosis and process monitoring of diseases [13,14]. Studies have shown that fluorescent labeling techniques allow the covalent bonding of fluorescent groups to attach to and recognize molecular substances, such as proteins and nucleic acids. Labeling using fluorescent probes can be covalent via chemical or enzymatic reactions, or non-covalent through binding equilibrium [15,16]. As a result, fluorescent labeling to visualize and monitor the surface of complex biological cell membranes has become one of the most prevalent methods for analyzing cellular functions and properties.

Nucleic acids, which include DNA and RNA, are a type of classic biological macromolecule widely used for storing and transmitting genetic information. DNA is the carrier and transmitter of genetic information in all living systems, consisting of four different deoxynucleotide monomers, as is common knowledge. Each monomer consists of a phosphate group, deoxyribose, and one of four nitrogenous nucleobases, while the nucleobases include thymine (T), adenine (A), guanine (G), and cytosine (C) [17,18]. Watson and Crick reported the double helix structure of DNA for the first time in 1953, leading to extensive research on DNA structure [19]. DNA, the traditional genetic molecule, has attracted a great deal of interest due to its exceptional sequence programmability, high molecular recognition accuracy, and numerous biological functions. Due to their biocompatibility, simple synthesis, ease of modification and functionalization, and modular structure, nucleic acid probes, particularly DNA probes, have been widely used over the past few decades. In addition, they can be combined with various signal amplification techniques to achieve additional functions [20,21,22]. According to the spatial dimension of nanostructures, the DNA nanomaterials commonly used in current research are mainly categorized into the typical assembling strategies of one-dimensional (1D), two-dimensional (2D), and three-dimensional (3D) nanostructures [21]. DNA-based nanomaterials and functional DNA sequences (Aptamer, DNAzyme, i-motif, G-quadruplex, etc.) endow DNA nanostructures with functions such as targeting, stimulating responses, and regulating life activities, which demonstrate unique advantages in disease diagnosis and cancer therapy [23,24]. The fluorescent labeling of DNA is a common technique used for many applications in bioanalysis and imaging. Environmentally sensitive fluorophores can sense the interaction of DNA with other (biological) molecules by altering some measurable property of the fluorescence (the intensity, wavelength, or lifetime) [16].

DNA nanostructure-based nucleic acid probes have shown great promise in biosensing, biological imaging, drug delivery, cell biology, and material manufacturing. The primary applications of DNA nanomaterials on the surfaces of cell membranes are fluorescence imaging and functional regulation. This article examines the use of DNA nanomaterials in the fluorescence imaging of cell membranes.

## 2. Application of Cell Membrane Imaging

### 2.1. Monitoring Imaging Triggered by the Tumor Cell Microenvironment

The tumor microenvironment (TME) is a physical and biochemical system that plays a significant role in the occurrence, development, metastasis, and drug resistance of tumors [25]. In general, the tumor microenvironment’s physiological state is distinct from that of normal tissue. Tumor tissue can be distinguished from normal tissue according to a number of physiological characteristics, including the overexpression of ATP, an acidic pH, hypoxia, a high level of reactive oxygen species (ROS), and the overexpression of enzymes. Consequently, these characteristics have become increasingly desirable as diagnostic and therapeutic targets [26,27]. 

#### 2.1.1. ATP

Adenosine triphosphate (ATP) is a fundamental biomolecule involved in numerous biochemical synthesis and metabolic processes. For a deeper understanding of the related cellular processes, it is crucial to examine the distribution of, and changes in, ATP outside the cell [28]. Previous research has demonstrated that the concentration of ATP in the extracellular environment of a tumor is 100–500 μM, which is 10^3^–10^4^ times greater than the concentration in the stroma of normal tissue (10–100 nM) [29]. To clarify the potential regulatory effects of extracellular ATP on various physiological activities, however, there are insufficient cell-surface fluorescent probes. Therefore, the development of simple and accurate fluorescent methods for the detection and localization of extracellular ATP on cell surfaces remains a key goal. 

**Figure 1:**
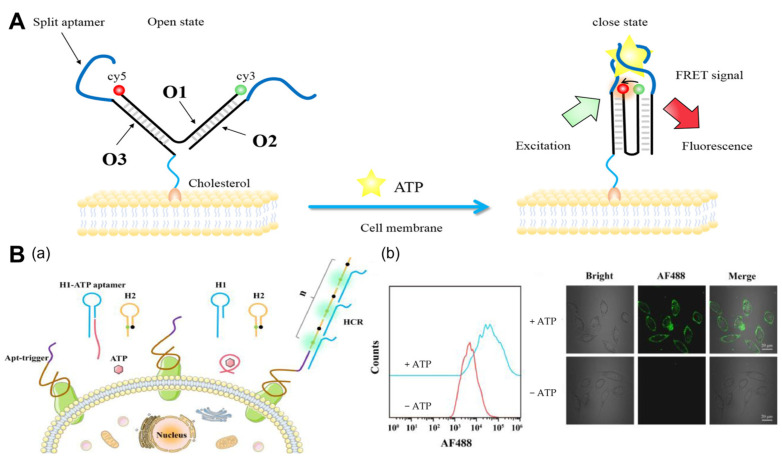
ATP imaging of the cell membrane. (**A**) Mechanism of the cell-surface anchored ratiometric DNA nanoswitch for the imaging of extracellular ATP [30]. The Apt-trigger probe consisted of two components: a ZYsls aptamer for specific binding to SMMC-7721 cells and a trigger sequence for initiating the HCR assembly. (**B**) (**a**) Schematic illustration of the extracellular ATP-activated hybridization chain reaction for cancer cell detection. (**b**) Flow cytometry assays and confocal fluorescence imaging of SMMC-7721 cells incubated with Apt-trigger/H1-ATP aptamer/H2 with or without the addition of ATP [34].

Utilizing DNA tweezers and cleavage aptamers, Zhong et al. [30] proposed a ratiometric DNA nanoswitch (Figure 1A). The nanoswitch consists of three uniquely designed ssDNA chains that are attached to the cell membrane via cholesterol. First, the DNA tweezers are in the open state, causing the fluorescent groups to separate and produce a low fluorescence resonance energy transfer (FRET) ratio. FRET is a mechanism that describes the transfer of energy between a photosensitive chromophore donor and an acceptor, and it is widely used in biochemistry and other areas [31]. Inducing the binding of the two split aptamers in the presence of ATP converts the nanoswitch from an open to a closed state, bringing the donor and acceptor closer together and generating a high FRET efficiency. This work has accomplished the imaging of cell membrane ATP with a simple design and lays the groundwork for the future performance of ATP imaging over an extended period of time. 

ATP imaging analysis alone is typically insufficient for sensitive analysis, so researchers frequently employ signal amplification for more sensitive ATP imaging. Catalytic hairpin assembly (CHA) and hybridization chain reaction (HCR) are two of the most widely used signal amplification strategies. The CHA is a reaction in which hairpin DNA can be activated by specific nucleic acid sequences and automatically forms a stable double-stranded body via thermodynamic entropy gain [32]. The HCR reaction requires the involvement of both hairpin DNAs, and in the presence of a target, one of the hairpin DNAs can be opened, which, in turn, triggers the opening of the other hairpin DNA to trigger the creation of the HCR, generating a long double-stranded tandem of DNA [33]. Wang et al. [34] proposed the signal amplification strategy of extracellular ATP-activated HCR amplification, which enables accurate and sensitive tumor cell detection. In complex biological matrices, this method has promising application potential. In addition, by altering the sequence of the corresponding aptamer, this method can be used to detect various types of tumor cells (Figure 1B). The extremely high concentration of ATP in extracellular TME not only promotes tumor cell extravasation and detachment from the primary site but also stimulates tumor cells and stromal cells to release matrix metalloproteinases (MMPs), such as MMP2/9. Li et al. [35] designed an intelligent DNA nanodevice that sequentially responds to MMP2/9 and ATP in the TME. This design will make multiple biomarkers visible concurrently and provide a thorough understanding of their pathological function in tumor metastasis. We summarize some of the different cellular tumor microenvironments for ATP imaging in Table 1.

#### 2.1.2. pH

**Figure 2:**
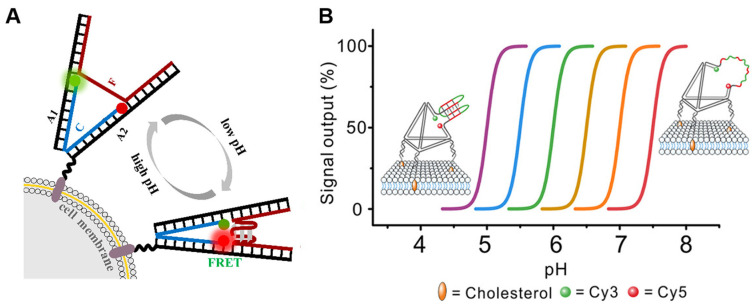
Acidic pH imaging of the tumor microenvironment. (**A**) Construction and principle of the pH-sensitive DNA tweezer [38]. The green ball is Rhodamine Green and the red ball is Rhodamine Red. DNA tweezer was anchored to the cell membrane by cholesterol. (**B**) The strategy to engineer programmable i-motif-TDF pH sensors [43].

In addition to the overexpression of ATP, extracellular pH (pHe) is a crucial microenvironmental factor in the development of tumors in the tumor microenvironment. The adjustment of pH value is essential for the maintenance of equilibrium in organisms. Several pathologies, such as ischemia, renal insufficiency, inflammation, and chronic lung disease, are typically associated with local pH fluctuations. Extracellular acidosis is becoming a universal indicator in the clinical diagnosis of tumors. To effectively distinguish normal cells from cancer cells, TME imaging must be sensitive. Nie et al. [38] designed a DNA tweezer composed of an i-motif to dynamically monitor pH changes in the cell microenvironment (Figure 2A). The sensor can respond to the extracellular pH quickly and reversibly in the pH range from 5.0 to 7.5 and image pH changes on the cell surface in real time with excellent temporal and spatial resolution. The same is true for DNA tweezer sensors. Jiang et al. [39] developed a novel DNA nanotweezer (NT) sensor based on a pH-responsive triplex–duplex conformation, which can achieve stable cell surface anchoring and the dynamic regulation of the extracellular pH value. The author’s nanomachine can serve a foundation for the use of pH sensing in the extracellular microenvironment and the diagnosis of various pH-related diseases. In order to achieve the accurate, highly sensitive, and high-resolution in situ detection of the cell surface pH value of living cells, Zhang et al. [40] designed a ratio fluorescent probe that aggregated when interacting with cells, enabling the continuous measurement of the cell surface pH value of labeled cells. The ternary nanoplatform is made up of bovine serum albumin-protected gold nanoclusters (BSA-AuNCs), cationic peptides (CPs) labeled with fluorescein isothiocyanate (FITC), and CPs without FITC. In the pH range from 5.0 to 9.5, the probe responds with a sensitive fluorescence ratio. 

Double-stranded DNA can be hydrolyzed; it is not a suitable candidate for sensor functions in biological media. In contrast, framework nucleic acids have an excellent resistance to enzymatic hydrolysis and are widely employed in numerous sensors [41,42]. Yuan et al. [43] designed a programmable pH sensor that employs the tetrahedral DNA framework (TDF) structure as the skeleton and the DNA i-motif structure as the proton recognition probe (Figure 2B). By modifying the sequence composition of the i-motif or combining it with different sensors, the response center and dynamic range of the i-motif to the pH can be precisely regulated to obtain a pH response window with excellent biological adaptability between 5.0 and 7.5. The use of framework nucleic acid structures for the backbone renders it more stable and means that it can be effectively anchored to the cell membrane. This is expected to provide a useful nanoplatform for the study of cell membrane imaging and functional regulation [44,45].

In addition, since the extracellular pH (pHe) of the tumor (6.5–6.8) is not significantly different from that of healthy tissue (7.4) [46], a signal amplification method with sufficient specificity and gain is required for acidic TME imaging. Li et al. [47] designed a method that combines extracellular acidity targeting and cell membrane engineering strategy with HCR-mediated signal amplification to achieve the sensitive imaging of acidic TME. By introducing a pH (low) insertion peptide for the selective surface labeling of tumor cells of artificial DNA receptors, the self-assembly of the fluorescent HCR amplifier was then triggered to amplify the weak acid signal. The experimental results demonstrated that HCR can be performed on the surface of cancer cells under acidic conditions using the strategy proposed by the authors. In addition to enhancing the detection sensitivity, this structure has the potential to facilitate selective drug delivery by affixing the target drug to the hairpin species.

#### 2.1.3. Metal Ions

**Figure 3:**
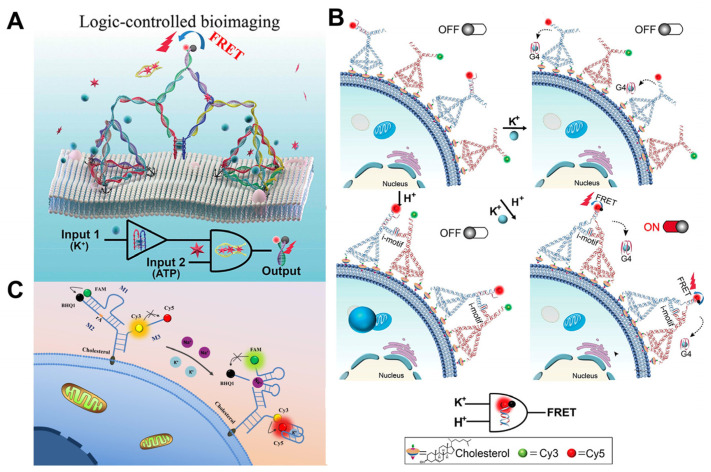
Tumor microenvironment logic gate imaging. (**A**) Logical imaging of tumor microenvironment K^+^ (blue ball) and ATP (red hexagon) [36]. (**B**) Logical imaging of tumor microenvironment K^+^ and H^+^ [53]. DNA tetrahedra were anchored to the cell membrane by cholesterol. (**C**) Sketch map showing the Y-shaped DNA sensor anchoring onto the cell surface to simultaneously monitor sodium and potassium in the cellular membrane microenvironment [49].

Some metal ions, such as sodium (Na^+^) and potassium (K^+^), play important roles in life processes, while ATP and pH have a wide variety of applications outside of cell surface imaging [48,49]. Cell surface K^+^ channels are assembled in the endoplasmic reticulum (ER) and transported through organelles to the plasma membrane [50]. In the process of rapid division, tumor cells release up to 50 mM of K^+^ into the extracellular space, thereby inhibiting the Akt-mTOR pathway and the activity of T-cell effector molecules, thus affecting their killing effect on T-cells, which causes cancer cells to escape the immune system and proliferate uncontrollably [51,52]. Extracellular potassium ions and ATP are found to be maintained at extremely high extracellular concentrations in the TME and are biomarkers for early cancer detection and tumor localization. DNA tetrahedral nanostructures are a typical class of DNA backbone nucleic acids with excellent mechanical rigidity, biocompatibility, and other advantages that have numerous potential applications in biology, biomedicine, and other fields. Taking advantage of this, Peng et al. [36] developed an intelligent DNA nano-assembly that used bimolecular G-quadruplex (G4) and the ATP aptamer as logic control units and was controlled by a “Yes and And” logic circuit to achieve the logical imaging of extracellular K^+^ and ATP, and thereby demonstrated its application potential in research into the mechanism of ATP-sensitive K^+^ channels. (Figure 3A) In addition, K^+^ and H^+^ can be used as input signals for logic gates in order to image cell membranes. Li et al. [53] designed the H^+^- and/or K^+^-responsive logic sensor, which was assembled by in situ dimer framework nucleic acid (FNA) on the cell surface, and applied the logic sensor for the first time to promote the intracellular internalization of molecular payload in an extracellular environment simulating a tumor (Figure 3B). The sensor immobilizes the anti-cancer aptamer AS1411 at the apex of the FNA, where the bimolecular i-motif is bound as a regulatory unit so that the dimer DNA nano-assembly can respond to the extracellular pH change. AS1411 was induced by K^+^ to fold into a G-quadruplex, which was then released from the dimer FNA, resulting in DNA hybridization-based FRET. Their team [54] continued to introduce pH-dependent triplexes into the stem-loop motifs of bimolecular G-quadruplexes (bi-G4s) based on this research. The resulting DNA topologies, triplex-enhanced G-quadruplexes (tb-G4s) underwent heterodimeric folding in the presence of K^+^ and pH 5.0, allowing them to direct the dimerization of the FNA nanoplatforms to which split ATP aptamers are attached. This provides a sensitive method for the dynamic assessment of drug-induced apoptosis and efficacy by monitoring apoptosis in real time by capturing endogenous ATP released during chemotherapeutic drug stimulation. 

Since Na^+^ and K^+^ ions play crucial roles in biological systems, Yang et al. [49] proposed a novel method for simultaneously analyzing Na^+^ and K^+^ in the cellular membrane microenvironment using a well-designed Y-shaped DNA sensor (Figure 3C). Single-ion analysis provides less comprehensive information than the simultaneous detection of Na^+^ and K^+^.

Other factors in the cell membrane microenvironment (such as the temperature [55] and gas signal molecules [56]) have gradually piqued the interest of researchers and are being increasingly utilized in fluorescence imaging.

### 2.2. Imaging of Cell Membrane Receptors

**Figure 4:**
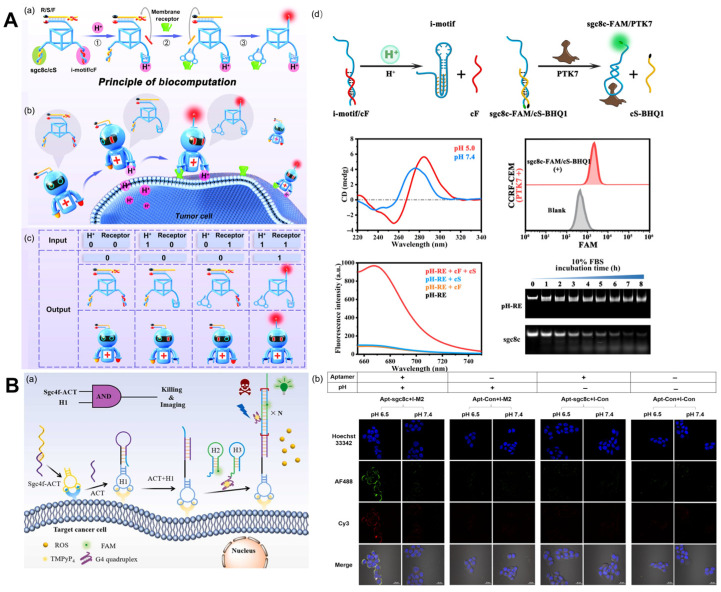
Cell membrane receptor imaging. (**A**) (**a**) Schematic of the molecular structure and operational mechanism of pH-RE. (**b**) Schematic of using pH-RE for in vivo precise tumor imaging. (**c**) The truth table for pH-RE-based DNA computation. (**d**) Schematic illustration of the pH-dependent displacement of a cF strand from an i-motif/cF module and some experimental results, including CD spectra of the pHe recognition module (2.5 μM) at different pH values. Recognition of PTK7 by the receptor recognition module (250 nM). Fluorescence change of pH-RE logic operation. Stability analysis of pH-RE and sgc8c ssDNA [68]. (**B**) (**a**) The working principle of the dual-activatable “molecular doctor” for the accurate imaging and killing of cancer cells. (**b**) Bispecific imaging of HCT116 cells via pH-Apt-MD [69].

Receptors are a class of protein molecules that serve as subcellular cell surfaces or intracellular components. Receptors on the cell membrane play important roles in a variety of physiological and pathological processes and have become therapeutic targets for a large number of drugs [57,58]. G-protein-coupled receptors (GPCRs) and enzyme-linked receptors (especially RTKs) are the two largest known receptors among the thousands of known cell surface receptors in eukaryotes, and they also represent one of the hottest topics in the field of life science [59]. Recognizing extracellular stimuli and transducing cell signal pathways, cell surface receptors regulate fundamental cell behaviors, such as proliferation, migration, and differentiation [60]. Numerous membrane receptors, such as MUC1 [61], nucleolin [57], EGFR [62], epithelial cell adhesion molecules [63], etc., have been considered tumor markers and used for the specific imaging of cancer cells up to this point. The majority of these receptors are RTKs and participate in numerous intracellular signaling pathways associated with the growth and proliferation of cancer cells [64]. Aptamers are commonly employed as protein-specific binding units for the detection of receptor proteins [65]. To detect receptor protein dimerization, Ouyang et al. [66] developed a dual-aptamer DNA probe with up-conversion nanoparticles and a photo-cleavage (PC) linker. When the probe reaches a particular cell position, the DNA aptamer probe is irradiated by ultraviolet light transformed from up-conversion nanoparticles, which causes the molecular chain to break, thereby producing a FRET signal (Figure 4A). Despite the widespread interest in the detection and imaging of cell membrane receptor proteins, identification based on a single biomarker may result in false-positive feedback, such as off-target phenomena due to the absence of tumor-specific antigens [67]. Through the dual-specific identification of the tumor cell microenvironment and cell surface receptors, in vivo imaging that is highly sensitive and highly responsive can generate new ideas for in vivo imaging. Li et al. [68] designed a DNA nanorobot that simultaneously detects extracellular pH and cancer cell membrane receptors (Figure 4A). This design employs two distinct types of biomarkers as inputs to accurately distinguish target tumor cells from similar cell mixtures; this could lead to novel approaches to the diagnosis and treatment of tumors.

Ingeniously, Wang et al. [69] designed an i-motif with a hairpin as an acid response element and paired it with a tumor-targeting nucleic acid aptamer for the bispecific imaging and in situ drug release of tumor cells, dubbing it the “molecular doctor” (pH-Apt-MD) (Figure 4B). It uses the binding of, and structural changes in, nucleic acid aptamers to the i-motif, the dissociation of pH-Apt-MD, and the stimulation of FRET signaling between AF488 and Cy3 to achieve in situ drug release. The integration of tumor diagnosis and treatment is a major direction of development for cancer treatment at present. The discovery of tumor markers and related aptamers enables the precise diagnosis and treatment of cancer. On the basis of this, Wang et al. [70] proposed a method of efficient signal amplification based on a DNA logic circuit that enables the accurate identification of tumor cells and photodynamic therapy. Aptamers Sgc4f and Sgc8c calculate cell membrane biomarkers using logic. Only when these two markers are present at the same time will HCR be activated and fluorescence be amplified.

**Figure 5:**
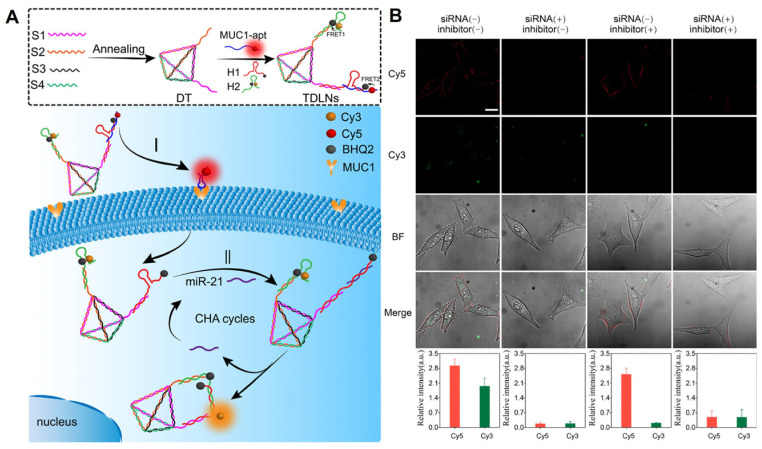
(**A**) Working principles of DNA-engineered logic nanodevices. (**B**) Evaluation of the dynamic change level of MUC1 and miR-21 in living cells via the TDLNs after different treatments [61]. MUC1 is distributed on the cell membrane and intracellular miR-21 restored Cy3 fluorescence.

As a method of diagnosing tumors, the imaging of receptors on cell membranes has been widely reported, but there may be false positives. In contrast, multiple or distinct tumor markers in living cells are crucial for obtaining precise and multidimensional information regarding tumor cell types and tumor progression. Consequently, using receptors on the cell membrane as logical input signals can prevent false-positive results and enable the rapid and sensitive transmembrane sequential imaging of multilayer biomarkers that precisely distinguish tumor cell types. For instance, our research team [61] incorporated a localized CHA reaction and DNA logic operation into DNA tetrahedron structures in order to distinguish cell types by monitoring MUC1 and miR-21 in living cells (Figure 5). These findings demonstrate that the newly developed DNA logic nanodevices have enhanced molecular targeting and recognition capabilities, allowing for the simple identification of cell types and the early diagnosis of cancer.

Yang et al. [71] have designed an alternative probe that uses the cell membrane receptor MUC1 to enter cells for subsequent miRNA imaging and gene silencing. The design may yield new insights for the early diagnosis and targeted treatment of tumors.

### 2.3. Imaging of Other Molecules

**Figure 6:**
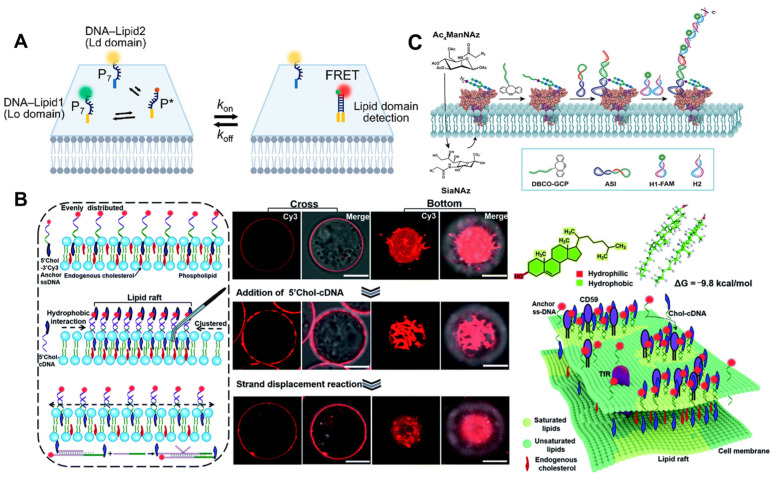
(**A**) Schematic of the DNA zipper system [74]. (**B**) Schematic illustrating the strategy of using DNA nanotweezers to manipulate the cholesterol distribution on a living cell membrane, which stabilizes and dynamically lights up lipid rafts [76]. Confocal fluorescence microscopy images of the cells focused on cross and bottom section and schematic illustrating the proposed strategy of using DNA nanotweezers to recruit raft-associated saturated lipids, membrane and possibility endogenous cholesterol. (**C**) Illustration of the amplified visualization of protein-specific glycosylation via proximity-induced HCR [79]. Target proteins are distributed across the cell membrane.

A complex mixture of lipids, proteins, and other components composes the cell membrane. Some lipids and proteins interact preferentially with other substances to form lipid domains. The plasma membrane adopts a heterogeneous structure model [72,73]. You et al. [74] reported several new types of lipid–DNA conjugates, termed “DNA zippers”, that can be used to measure the dynamic interactions of cell membranes and the formation of lipid domains in order to study cell membrane lipid domains (Figure 6A). Several DNA probes for substance interactions at cell membranes may also have been reported, contributing to our knowledge of the structure and function of cell membranes.

The lipid raft hypothesis proposes that transient nanodomains with a high concentration of sphingolipids, sterols, and specific proteins exist on the cell membrane [72,75]. Li et al. [76] comprised a DNA nanotweezer composed of a cholesterol-functionalized DNA duplex, which can stabilize short-lived lipid rafts in order to comprehend the potential relationship between lipid rafts and cellular functions. Other spectral tools are useful for further analyzing the components and functions of lipid rafts (Figure 6B). The author’s proposed DNA nanotweezers can induce the aggregation of raft-related components (saturated lipids, membrane proteins, and possibly endogenous cholesterol) and lead to T cell proliferation via the aggregation of T cell antigen receptor (TCR). The editable nature of DNA-based nanomaterials will create new avenues for T-cell therapies in the future. Ju et al. [77] developed a hierarchical fluorescence imaging strategy with N- N-acetylneuraminic acid (Sia) as the model sugar by combining specific raft recognition, chemical selective labeling of polysaccharide, and DNA dynamic hybridization technology to achieve the simultaneous visualization of raft and raft-harbored glycans on the cell surface. Consequently, this strategy contributes to the discovery of the glycan regulation mechanisms on rafts. Recently, several FRET-based methods of studying protein-specific glycans have been developed. In addition to selective labeling, it is necessary to overcome the relatively low sensitivity of FRET-based methods [78]. Thus, Yang et al. [79] demonstrated a proximity-induced HCR strategy for visualizing and magnifying protein-specific glycosylation. It was utilized to observe tyrosine-protein kinase 7-specific sialic acid in living CEM cells and zebrafish, as well as to track its changes during drug treatment (Figure 6C). It is a potential tool for studying the specific glycosylation of proteins and the relationship between the dynamic glycan state and the disease process.

Moreover, sialylation is essential for numerous physiological processes, and abnormal salivation is closely associated with the development of disease [80]. Particularly during the onset and progression of cancer, the alteration of the sialylation state on the surface of specific cells represents a wealth of cancer-related data. Among the numerous imaging tools, the FRET technique is an ingenious approach upon which researchers have devised a series of strategies for imaging sialylation acidification on specific cell surfaces [81,82].

## 3. Conclusions and Outlook

The study of cell membranes has attracted the attention of a significant number of scientists in recent years and warrants further investigation in the future. Recent applications of DNA nanomaterials in the fluorescence imaging of cell membranes, including the imaging of tumor microenvironment, cell membrane receptors, and other molecules, are discussed in this review. Because of the excellent programmability, great biocompatibility, and easy modification of DNA nanomaterials, an increasing number of nanodevices are being used for the fluorescence imaging of cells, an approach that has significant biomedical potential. No only can the DNA probe enter the cell for imaging via endocytosis [83] but it can also be anchored on the cell membrane for the imaging of the cell membrane [84].

Although DNA nanomaterials are widely employed in cell surface fluorescence, the following obstacles must still be addressed. (1) Nowadays, most current imaging strategies involve imaging and analyzing only one or two targets, but using signal amplification techniques, such as HCR and CHA, to achieve the in situ accurate and highly sensitive imaging of multiple targets in the tumor microenvironment remains a challenge. (2) Aptamers must be designed specifically for each receptor. Currently, SELEX technology is used to screen all aptamers, and there are still receptors without aptamers. (3) The majority of DNA nanomaterials are attached to the cell membrane via covalent or noncovalent methods [44,85] but are susceptible to endocytosis. Even though some scientists have developed polymer molecular skeletons to increase the anchoring time of probes in cell membranes, long-term stability remains a problem. Monitoring intra- and extracellular signaling, cellular morphology, and structural changes requires prolonged in situ imaging, especially for slow-response events such as apoptosis. The creation of probes that can remain attached to the cell membrane for extended durations without being endocytosed remains an area of active research. (4) DNA nanomaterials are used not only for the fluorescence imaging of cell membranes but also for the functional regulation of cell membrane receptors. How to realize the integration of long-term imaging and the functional regulation of cell membranes should also be investigated. (5) FRET is dependent not only on the donor (D)–acceptor (A) separation distance and energetic resonance (i.e., D–A spectral overlap), but also on the orientation of the D emission and A absorption transition dipole moments [86,87]. How to enhance the intensity and duration of DNA nanomaterials based on cell membrane imaging by controlling the dipole orientation in FRET pairs is an area worth exploring.

With the advancement of DNA nanotechnology, an increasing number of DNA nanomaterials will be used for the fluorescence imaging of cell membranes, which will aid in our understanding of the occurrence and progression of disease and will have significant potential in biosensing, imaging analysis, treatment, and diagnosis.

## Figures and Tables

**Table 1:** Summary of the ATP imaging of the cell membrane.

Target	Linear Range	Cell Type	References
ATP	0–1000 μM	MDA-MB-231	[29]
ATP	5–60 μM	A549	[30]
ATP	10–100 nM	SMMC-7721	[34]
ATP	0–500 μM	HeLa	[36]
ATP	0–5 mM	HeLa	[37]

## Data Availability

Not applicable.
